# Downregulation of 14-3-3σ Correlates with Multistage Carcinogenesis and Poor Prognosis of Esophageal Squamous Cell Carcinoma

**DOI:** 10.1371/journal.pone.0095386

**Published:** 2014-04-17

**Authors:** Yi-Jun Qi, Ming Wang, Rui-Min Liu, Hua Wei, Wei-Xia Chao, Tian Zhang, Qiang Lou, Xiu-Min Li, Jin Ma, Han Zhu, Zhen-Hua Yang, Hai-Qing Liu, Yuan-Fang Ma

**Affiliations:** 1 Key Laboratory of Cellular and Molecular Immunology, College of Medicine, Henan University, Kaifeng, Henan, P. R. China; 2 Huaihe Hospital, Henan University, Kaifeng, Henan, P. R. China; 3 Xinxiang Medical University, Xinxiang, Henan, China; 4 Linzhou Cancer Hospital, Linzhou, Henan, P. R. China; National Cancer Institute, National Institutes of Health, United States of America

## Abstract

**Aims:**

The asymptomatic nature of early-stage esophageal squamous cell carcinoma (ESCC) results in late presentation and consequent dismal prognosis This study characterized 14-3-3σ protein expression in the multi-stage development of ESCC and determined its correlation with clinical features and prognosis.

**Materials and Methods:**

Western blot was used to examine 14-3-3σ protein expression in normal esophageal epithelium (NEE), low grade intraepithelial neoplasia (LGIN), high grade intraepithelial neoplasia (HGIN), ESCC of TNM I to IV stage and various esophageal epithelial cell lines with different biological behavior. Immunohistochemistry was used to estimate 14-3-3σ protein in 110 biopsy samples of NEE, LGIN or HGIN and in 168 ESCC samples all of whom had follow-up data. Support vector machine (SVM) was used to develop a classifier for prognosis.

**Results:**

14-3-3σ decreased progressively from NEE to LGIN, to HGIN, and to ESCC. Chemoresistant sub-lines of EC9706/PTX and EC9706/CDDP showed high expression of 14-3-3σ protein compared with non-chemoresistant ESCC cell lines and immortalized NEC. Furthermore, the downregulation of 14-3-3σ correlated significantly with histological grade (*P* = 0.000) and worse prognosis (*P* = 0.004). Multivariate Cox regression analysis indicated that 14-3-3σ protein (*P* = 0.016) and T stage (*P* = 0.000) were independent prognostic factors for ESCC. The SVM ESCC classifier comprising sex, age, T stage, histological grade, lymph node metastasis, clinical stage and 14-3-3σ, distinguished significantly lower- and higher-risk ESCC patients (91.67% vs. 3.62%, *P* = 0.000).

**Conclusions:**

Downregulation of 14-3-3σ arises early in the development of ESCC and predicts poor survival, suggesting that 14-3-3σ may be a biomarker for early detection of high-risk subjects and diagnosis of ESCC. Our seven-feature SVM classifier for ESCC prognosis may help to inform clinical decisions and tailor individual therapy.

## Introduction

Esophageal squamous cell carcinoma (ESCC) accounts for nearly 90% of all esophageal cancers and is the fourth leading cause of cancer death in China [1]. Despite significant diagnostic and therapeutic advances, the 5-year overall survival rate for ESCC is still less than 25%, due mainly to distant metastasis and limited therapeutic options at initial diagnosis [2], [3]. In sharp contrast, the 5-year survival rate for ESCC patients at early stages is more than 90% [4], [5]. Although a variety of molecular alterations have been identified over the last two decades, sensitive and specific biomarkers for early diagnosis and accurate indicators for ESCC prognosis are currently unavailable. It is imperative, therefore, to identify novel biomarkers for early detection, and therapeutic targets if long-term survival of ESCC is to be improved.

The 14-3-3 proteins comprise a family of highly conserved small acidic proteins expressed in all eukaryotic organisms. In mammals, seven isoforms (α/β, γ, ε, σ, ζ, τ/θ and η) [6], [7], are implicated in diverse biological processes including protein trafficking, metabolism, cell cycle progression, cell differentiation, senescence, apoptosis, DNA repair and malignant transformation [8]–[11]. Of these seven mammalian isoforms, 14-3-3σ is uniquely expressed in epithelial cells and is linked most directly to cancer [8], [12]. Because it is a negative regulator of cell cycle and since there is reciprocal modulation between 14-3-3σ and p53, 14-3-3σ has been suggested as a potential tumor suppressor [8], [13]. Recently, downregulation of 14-3-3σ has been reported in various cancers of epithelial origin, including breast [14]–[21], lung [22], colon [23], liver [24], stomach [23], prostate [25]–[27], ovary [28]–[30], nasopharynx [31], oral cavity [32], ESCC [33], head and neck [34]. Conversely, overexpression of 14-3-3σ has also been observed in many cancers, including pancreas [35]–[37], colorectal [38], head and neck [39], lung [40], [41] and ESCC [42]. Furthermore, the correlation of 14-3-3σ and prognosis varies in different malignancies. It is likely, therefore, that the role of 14-3-3σ in human carcinogenesis is context-dependent. In the case of ESCC, more studies are needed to characterize the expression of 14-3-3σ during the multi-stage disease development and its prognostic value.

In this study, we investigated the expression pattern of 14-3-3σ in biopsy and resected ESCC, and evaluated its relationship with clinicopathological features and survival.

## Materials and Methods

### Clinical Specimens and Cell Lines

This study was approved by the Ethnics Committee of the Medical School, Henan University, China. All patients gave informed consent prior to sample collection. The first cohort of fresh ESCC samples, including 52 male and 28 female patients with a median age of 63.8 years (range, 47–69 years), were collected from Linzhou Cancer Hospital, Henan, China, between 2010 and 2011. Primary tumor and matched distal normal esophageal mucosa were separated by an experienced pathologist and snap-frozen in liquid nitrogen immediately following surgical resection and stored at −70°C. The second cohort of samples comprised fresh biopsies collected from Huaihe Hospital, Henan University, China and formalin-fixed biopsies collected from Linzhou Cancer Hospital, China between 2010 and 2012. Of the 50 fresh biopsy samples, 20 were normal esophageal epithelium (NEE), 17 mild and/or moderate dysplasia (defined as low grade intraepithelial neoplasia (LGIN)), 13 severe dysplasia and/or carcinoma *in situ* (defined as high grade intraepithelial neoplasia (HGIN)) [43], and of the 60 formalin-fixed biopsy samples, 21 were NEE, 20 LGIN, 19 HGIN. 14-3-3σ expression was studied retrospectively in formalin-fixed, paraffin embedded archival specimens of 82 primary ESCC patients undergoing surgery at Huaihe Hospital, Henan University, China between 2003 and 2008, and an ESCC tissue microarray (TMA, Shanghai Outdo Biotech Co., Ltd.) comprising 86 ESCC patients undergoing surgery between 2006 and 2008. Of the 168 ESCC patients, 125 were male and 43 female (median age of 67.0, range 51–80 years). The median follow-up period was 42.3 months (range, 1–60 months). None of ESCC patients received radiotherapy or chemotherapy before surgery. The clinical stage of all ESCC patients was classified or reclassified according to the seventh edition of the American Joint Committee on Cancer staging system. The fresh biopsy samples were stored in liquid nitrogen or at −70°C, and the archival samples of biopsy and ESCC were formalin-fixed, paraffin embedded and stored at room temperature until further analysis. The clinicopathological features for each cohort samples are summarized in Table S1.

An immortalized esophageal epithelial cell line (NEC), three ESCC cell lines (EC1, EC109, EC9706), a paclitaxel-resistant sub-line EC9706/PTX and a cisplatin-resistant sub-line EC9706/CDDP derived from parental EC9706 cell line were maintained in RPMI1640 supplemented with 10% fetal bovine serum, 100 units/ml penicillin G, and 100 ug/ml streptomycin at 37°C in a 5% CO_2_ incubator.

### Western Blot

For Western blot analysis, we used 50 frozen biopsy samples from Huaihe Hospital and 80 pairs of ESCC with matched NEE from Linzhou Cancer Hospital (20 pairs from each stage). For preparation of protein samples, chopped tissue samples were homogenized in lysis buffer (8 M urea, 4% CHAPS, 40 mM DTT) supplemented with complete proteinase inhibitor cocktail (Roche) on ice for 30 min. The lysates were centrifuged at 13,000 rpm for 15 min at 4°C to remove insoluble material and the supernatant collected and stored at −80°C until further use. After protein concentration measurement by the Bradford assay, 40 ug protein per sample were separated by 12% SDS-PAGE and transferred onto a PVDF membrane. The blots were blocked with 5% skimmed milk in TBST (10 mM Tris-HCl, 150 mM NaCl, 0.1% Tween, pH 7.4) for 1 hr at room temperature and then incubated with monoclonal mouse anti-14-3-3σ primary antibody (1∶5000, sc-100638, Santa Cruz, USA) overnight at 4°C followed by incubation with horseradish peroxidase-conjugated goat anti-mouse antibody (1∶10000, P0447, Dako, USA) for 1 hr at room temperature. The signal was visualized using enhanced chemiluminescence reagent (NCI5080, Thermo, USA). β-actin was detected simultaneously using anti-β-actin antibody (1∶5000, sc-47778, Santa Cruz, USA) as a loading control. Protein bands were quantified by densitometry using Quantity One software (Bio-Rad, USA).

### Immunohistochemistry (IHC) and Scoring of Immunostaining

We used 110 biopsy samples, samples for 82 ESCC patients with adjacent non-tumor NEE and an ESCC tissue microarray (86 ESCC patients) for IHC analysis of 14-3-3σ expression. Briefly, 5 µm-thick tissue sections were deparaffinized, rehydrated, incubated with 3% hydrogen peroxide solution for 10 min, heated in antigen retrieval solution EDTA buffer (10 mmol/L Tris Base, 1 mmol/L EDTA, pH 9.0) for antigen recovery, blocked with normal non-immune mouse serum for 20 min and then incubated with a mouse monoclonal antibody against 14-3-3σ (1∶200, sc-100638, Santa Cruz, USA) overnight at 4°C. Biotinylated secondary antibody against mouse IgG was applied for 1 hr at room temperature followed by ABC (PK4001, Vector Laboratories, USA) solution for 1 hr. Signal was visualized with diaminobenzidine (DAB, ZLI-9032, ZSBIO, China), counterstained with hematoxylin, dehydrated in ethanol, cleared in xylene and mounted.

IHC results were evaluated and scored independently by two investigators with no prior knowledge of the clinicopathological data of the patients. Semi-quantitative estimation for each slide or core was performed using composite scores by multiplying the staining intensity and positivity scores (overall score range, 0–12). The cytoplasmic and/or nuclear staining intensity was graded as 0 (absent), 1 (weak), 2 (moderate), 3 (strong) and the percentage of positively stained cells was graded as 0 (<10% positive cells), 1 (10–25%), 2 (26–50%), 3 (51–75%), 4 (76–100%) [44]. The composite score for each patient was further simplified by dichotomizing it to low expression (overall score of ≤6) or high expression (score of ≥7).

### Prognostic Prediction using Support Vector Machine (SVM)-based Methods

SVM is a powerful analytical tool for data classification and function approximation [45]. To improve the accuracy of prognostic prediction based on clinicopathological parameters together with 14-3-3σ expression, a SVM based prognostic model was generated. In this approach, 8 ESCC patients were excluded due to incomplete information and 160 ESCC patients were randomly divided into 120 patients used for SVM training and 40 patients for performance assessment of the SVM classifier. The radial basis function kernel was used to construct a non-linear SVM classifier. Leave-one-out (LOO) cross validation was used to determine the optimal values of the kernel parameter σ and regularization parameter C, and the test error was obtained using the tuned parameters. The performance of SVM was estimated using LOO cross validation error. All combinations of the 14-3-3σ immunostaining score and clinicopathological parameters including sex, age, T stage, histological grade, lymph node metastasis, clinical stage, were used to develop the best SVM model for prognostic prediction. The programs were coded using Matlab software.

### Statistical Analysis

All statistical analyses were performed with SPSS 16.0 software (SPSS, Chicago, IL, USA). Data are expressed as mean ± standard deviation (SD). Mann-Whitney tests or Wilcoxon signed-rank tests were used to evaluate the significance of the differences in 14-3-3σ expression normalized to β-actin. Survival was calculated from the date of surgery to the date of last follow-up or death. Patients that died from ESCC were considered as uncensored whereas surviving patients at the end of follow-up interval and patients dying from causes other than ESCC were regarded as censored data. Survival curves were estimated by Kaplan-Meier method and differences between curves were tested by log-rank tests. The significance of prognostic factors on survival was studied by Cox regression model. Receiver operating characteristic (ROC) curve analysis was used to estimate the predictive values of the clinicopathological parameters and SVM classifier. The chi-square test or Fisher’s exact test were used to evaluate the associations between 14-3-3σ expression and clinicopathological features. The gamma test was performed to evaluate the relationship between 14-3-3σ expression and ESCC progression and included NEE, LGIN, HGIN and ESCC together with the various clinical stages. *P*<0.05 was considered statistically significant.

## Results

### Downregulation of 14-3-3σ Protein in Precancerous Lesions and ESCC

**Figure 1:**
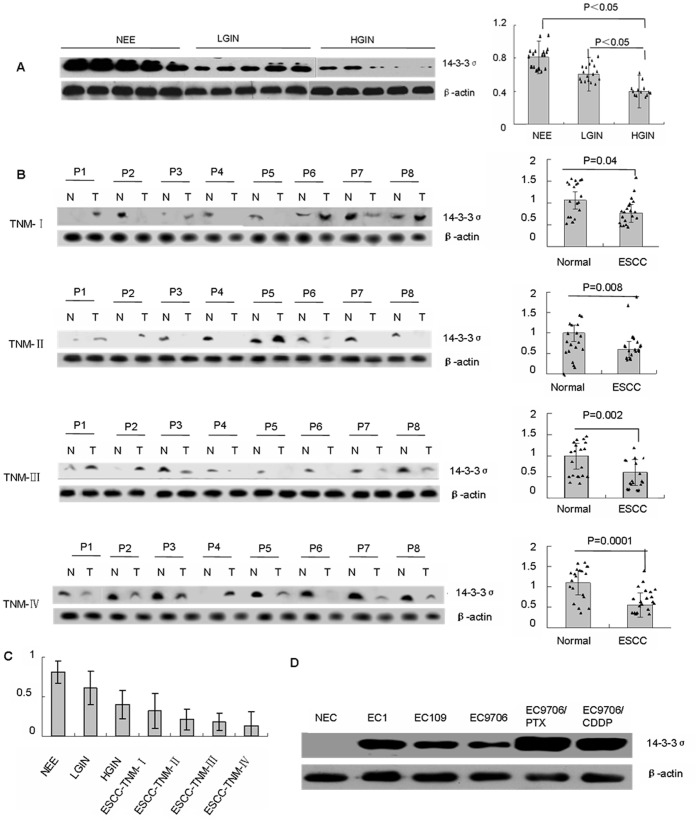
Western blot analysis and quantification of 14-3-3σ protein levels in different tissue types and esophageal epithelial cell lines. (A) Representative Western blot of 14-3-3σ protein levels and quantification in 20 samples of normal esophageal epithelia (NEE), 17 samples of low grade intraepithelial neoplasia (LGIN) and 13 samples of high grade intraepithelial neoplasia (HGIN). (B) Representative Western blot of 14-3-3σ protein levels and quantification in 80 paired samples of ESCC (T) and adjacent normal esophageal epithelia (N) with various clinical stages (20 ESCC samples for each stage). (C) Quantification of 14-3-3σ protein levels in the continuum of ESCC progression. (D) 14-3-3σ protein levels in an immortalized primary esophageal epithelial cell line NEC, three ESCC cell lines EC1, EC109 and EC9706, one paclitaxel-resistant cell line EC9706/PTX and one cisplatin-resistant cell line EC9706/CDDP. β-actin was used as a control for equal loading.

**Figure 2:**
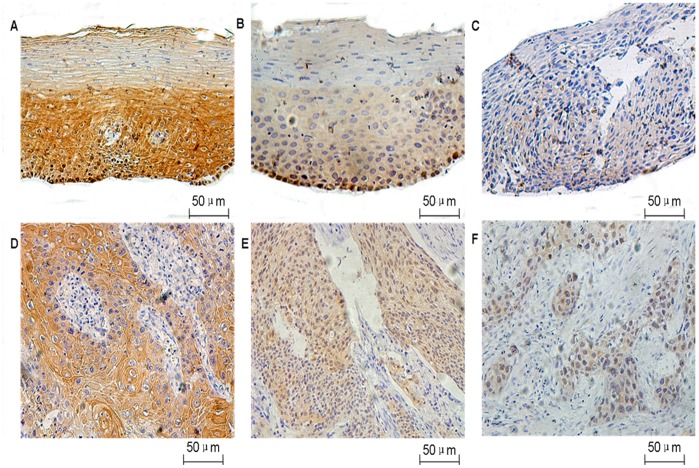
Representative immunohistochemical staining of 14-3-3σ. Normal esophageal epithelia (A), low grade intraepithelial neoplasia (B), high grade intraepithelial neoplasia (C), well-differentiated (D), moderately-differentiated (E) and poorly-differentiated ESCC (F).

14-3-3σ protein was measured in precancerous lesions and ESCC at various clinical stages. Western blots of precancerous lesions showed that 14-3-3σ protein was attenuated in a stepwise manner from NEE to LGIN, and to HGIN, and reached its lowest level in HGIN (Figure 1A). In addition, downregulation or loss of 14-3-3σ protein expression in ESCC at different clinical stages occurred in a pattern similar to the development from NEE to precursors and subsequently to ESCC. Lower level of 14-3-3σ protein expression correlated with later clinical stages (Figure 1B & C). Consistent with the Western blot analysis of 14-3-3σ alteration during the multi-stage development of ESCC, IHC analysis showed that the frequency of 14-3-3σ protein expression was greatest in NEE (77.5%) and decreased gradually during the evolution of esophageal carcinogenesis, with only 26.2% (44/163) of ESCC showing high expression of 14-3-3σ protein (Table 1, Figure 2B). Furthermore, a χ^2^ test showed that there was a significant difference when comparing the prevalence of 14-3-3σ suppression in various levels of cancer progression (*P* = 0.000). In-depth analysis revealed that 14-3-3σ protein was decreased significantly in HGIN and ESCC compared with NEE (*P* = 0.000) and there were no significant differences between NEE and LGIN, LGIN and HGIN, HGIN and ESCC (*P*>0.05, Table 1). The trend test using Gamma test indicated that ESCC progression negatively correlates with 14-3-3σ expression (Gamma value = -6.163, *P* = 0.000), suggesting that 14-3-3σ suppression may enhance the development and progression of ESCC. There was no difference with regards to staining pattern and the frequency of 14-3-3σ expression between biopsy and adjacent non-tumor mucosa of resected ESCC, and between biopsy of various tissue types collected from Huaihe Hospital and Linzhou Cancer Hospital as well (data now shown).

**Table 1:** 14-3-3σ protein expression during cancer progression by IHC analysis.

Cancer progression	Immunostaining		P
	Low	High	
NEE^ 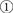 ^	9(25.5%)	31(77.5%)	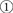 : 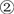 P = 0.087 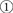 : 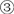 P = 0.000
Low grade intraepithelial neoplasia^ 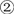 ^	16(43.2%)	21(56.8%)	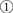 : 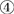 P = 0.000 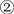 : 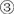 P = 0.090
High grade intraepithelial neoplasia^ 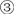 ^	21(65.6%)	11(34.4%)	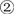 : 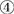 P = 0.001 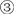 : 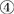 P = 0.389
ESCC^ 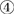 ^	124(73.8%)	44(26.2%)	

Note: Low grade intraepithelial neoplasia comprises mild & moderate dysplasia; High grade intraepithelial neoplasia comprises severe dysplasia & carcinoma in situ.

The 14-3-3σ protein level was also determined in an immortalized primary esophageal epithelial cell line (NEC), three ESCC cell lines (EC1, EC109 and EC9706), a paclitaxel-resistant sub-line (EC9706/PTX) and a cisplatin-resistant sub-line (EC9706/CDDP). NEC showed undetectable expression of 14-3-3σ protein which may contribute to the immortalized phenotype. However, three ESCC cell lines all expressed moderate levels of 14-3-3σ protein and the chemoresistant cell lines EC9706/PTX and EC9706/CDDP showed a higher expression of 14-3-3σ protein when compared with non-chemoresistant ESCC cell lines and immortalized NEC. Nevertheless, expression of 14-3-3σ protein in these cell lines was still lower compared with NEE (data not shown).

### Association between 14-3-3σ Expression and Clinicopathological Parameters of ESCC

Representative immunostaining of 14-3-3σ in different tissue types is shown in Figure 2. 14-3-3σ immunostaining was located predominantly in the cytoplasm and plasma membrane, and to a lesser extent in nucleus. In NEE, 14-3-3σ was strongly expressed in the nuclei of the majority of basal cells, in the cytoplasm and membrane of suprabasal cells but was present at only very low levels in the superficial layers in NEE. During the progression of ESCC from high to low histological grade, moderate to poor differentiation, ESCC showed increased nuclear staining of 14-3-3σ although the overall staining was significantly decreased compared with NEE. We further determined the association between 14-3-3σ expression and clinicopathological parameters of ESCC using IHC analysis of ESCC with various stages of ESCC (Table 2). Expression of 14-3-3σ was negatively correlated with histological grade (well- vs. moderate- and poor-differentiation, *P* = 0.000), whereas there was no significant correlation with age, gender, primary tumor sites, TNM stage or lymph node metastasis. The staining pattern and frequency of 14-3-3σ expression were not related sample origin.

**Table 2:** Association between 14-3-3σ expression and clinicopathological parameters of ESCC.

Variable		IHC expression		
		Low expression (n = 124)	High expression (n = 44)	P
Age	<60	38(22.6%)	17(10.1%)	0.354
	≥60	86(51.2%)	27(16.1%)	
Gender	Male	94(56.0%)	31(18.5%)	0.547
	Female	30(17.9%)	13(7.7%)	
Histological grade*	Well differentiation	12(7.1%)	16(9.5%)	0.000
	Moderate differentiation	73(43.5%)	18(10.7%)	
	Poor differentiation	39(23.2%)	10(6.0%)	
T stage	T1+T2	22(13.8%)	10(6.3%)	0.368
	T3+T4	98(61.3%)	30(18.8%)	
Lymph node metastasis	No	71(43.3%)	26(15.9%)	0.719
	Yes	51(31.1%)	16(9.8%)	
TNM stage	I–II	73(46.8%)	24(15.4%)	1.0
	III–IV	45(28.9%)	14(9.0%)	

*Well-differentiation vs. Moderate differentiation+Poor differentiation.

### 14-3-3σ Downregulation Predicts Poor Survival of ESCC

**Figure 3:**
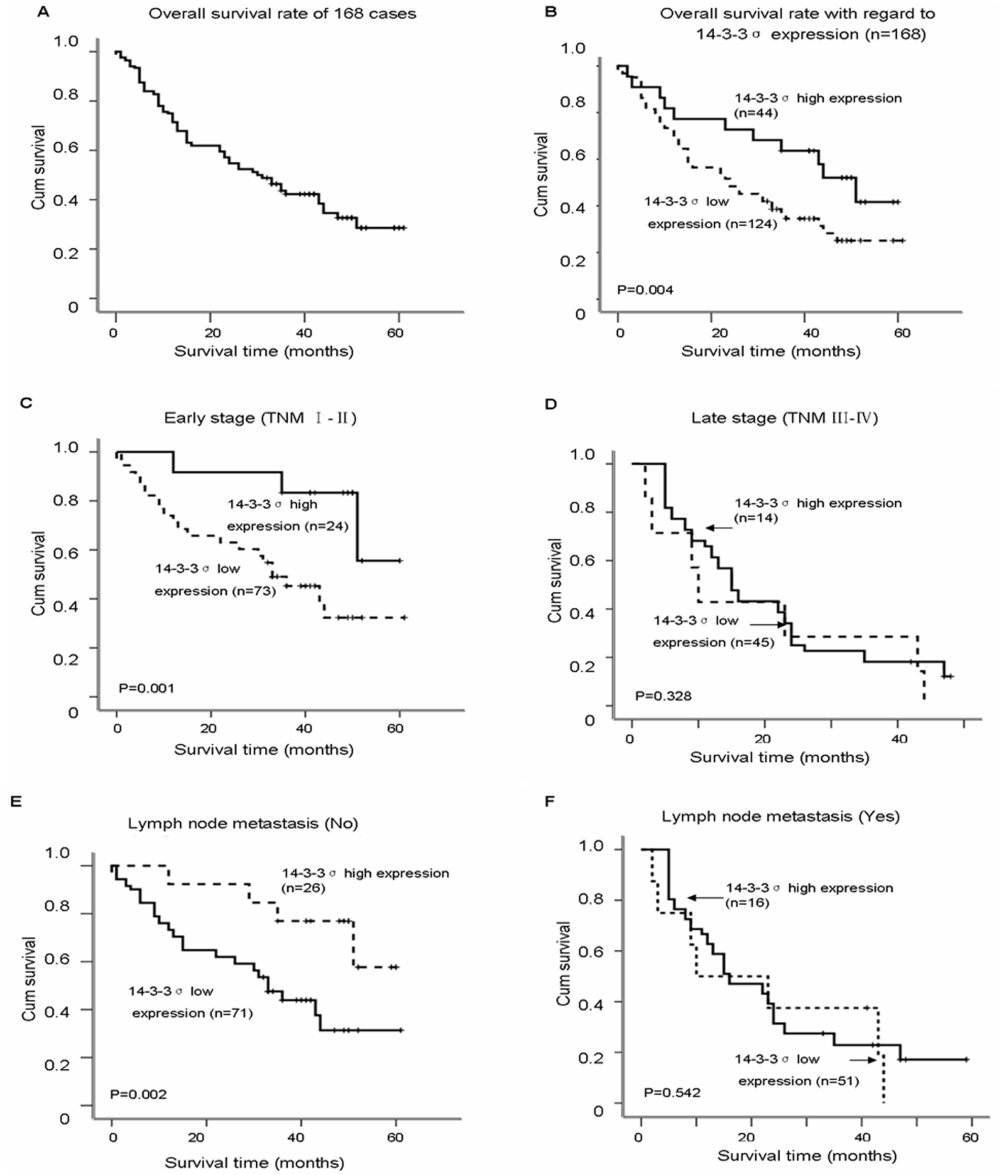
Kaplan-Meier survival curves of ESCC patients. (A) The 5-year overall survival rate of 168 ESCC patients was 30.26%. (B) The 5-year overall survival rates in ESCC patients with low (n = 124) and high 14-3-3σ protein (n = 44) were 25.62% and 43.47%, respectively, with a significant difference (*P* = 0.004). (C) The 5-year overall survival rates were 64.66%, 33.35% in ESCC patients with high (n = 24) and low expression (n = 73) of 14-3-3σ, respectively in early stage (I–II) ESCC; there was a significant difference in the overall survival rate between the two groups (*P* = 0.001). (D) No significant differences in 5-year survival rates were found between low levels (n = 45) and high levels (n = 14) of 14-3-3σ expression in ESCC patients with late clinical stage (III–IV, *P* = 0.328). (E) The 5-year overall survival rates in ESCC patients without lymph node metastasis were 61.28%, 32.54% in high (n = 26) and low levels (n = 71) of 14-3-3σ expression, respectively; there was a significant difference in the overall survival rate between the two groups (*P* = 0.002). (F) No significant differences in 5-year survival rates were found between low levels (n = 51) and high levels (n = 16) of 14-3-3σ expression in ESCC patients with lymph node metastasis (*P* = 0.542).

The association between 14-3-3σ protein expression and overall survival of ESCC was estimated using log-rank test and multivariable Cox proportional hazard regression analysis. The 5-year overall survival rate for all 168 ESCC patients was 30.26% (Figure 3A). Kaplan-Meier survival analysis showed that ESCC patients with low or loss expression of 14-3-3σ protein had a significantly worse prognosis than ESCC with higher expression. The 5-year overall survival rate in ESCC patients with low (n = 124) and high 14-3-3σ protein (n = 44) were 25.62% and 43.47%, respectively (log-rank test, χ^2^ = 8.448, *P* = 0.004, Figure 3B). In addition, early stage ESCC patients (stage I–II, 40.57%, n = 97) survived longer than later stage ESCC cases (Stage III–IV, 8.14%, n = 67, log-rank test, χ^2^ = 27.745, *P* = 0.000, data not shown). When the ESCC patients were stratified according to clinical stage, the 5-year overall survival rate was 64.66%, 33.35% in ESCC patients with high and low expression of 14-3-3σ, respectively in early stage ESCC (log-rank test, χ^2^ = 11.168, *P* = 0.001, Figure 3C), whereas 14-3-3σ was not related to ESCC survival in later stage ESCC (log-rank test, χ^2^ = 0.957, *P* = 0.328, Figure 3D). ESCC patients without lymph node metastasis (39.78%) survived longer than patients with lymph node metastasis (14.21%, log-rank test, χ^2^ = 18.726, *P* = 0.000, data not shown). When the patients were stratified by lymph node metastasis, those with high expression of 14-3-3σ had better survival (61.28%) than those with low 14-3-3σ expression (32.54%) in ESCC without lymph node metastasis (log-rank test, χ^2^ = 10.023, P = 0.002, Figure 3E), whereas 14-3-3σ expression was not correlated with lymph node metastasis (log-rank text, χ^2^ = 0.117, *P* = 0.732, Figure 3F).

To identify independent prognostic factors for ESCC survival, univariate and multivariate Cox regression models were performed. Univariate Cox proportional hazard regression analysis revealed that gender (Hazard ratio = 0.472, 95% CI = 0.284–0.784, *P* = 0.004), T stage (Hazard ratio = 0.430, 95% CI = 0.274–0.676, *P* = 0.000), N stage (Hazard ratio = 2.254, 95% CI = 1.533–3.313, *P* = 0.000), clinical stage (Hazard ratio = 2.741, 95% CI = 1.841–4.081, *P* = 0.000), and 14-3-3σ (Hazard ratio = 0.508, 95% CI = 0.316–0.816, *P* = 0.005) were significant prognostic predictors for overall survival of ESCC patients (Table 3). Other clinicopathological parameters including histological grade and age were not prognostic factors for the overall survival in our study (Table 3). Furthermore, multivariate Cox proportional hazards regression analysis indicated that T stage (Hazard ratio = 0.388, 95% CI = 0.241–0.623, *P* = 0.000) and 14-3-3σ protein (Hazard ratio = 0.466, 95% CI = 0.251–0.866, *P* = 0.016) were independent prognostic factors of ESCC (Table 3).

**Table 3:** Univariate and multivariate Cox regression analyses of the prognostic variables in ESCC patients.

Variables	Subset	Hazard ratio	95% CI	P
Univariate analysis(n = 168)				
Age	<60 vs. ≥60	1.390	(0.924–2.091)	0.114
Gender	Male vs. Female	0.472	(0.284–0.784)	0.004
T-stage	T1+T2 vs. T3+T4	2.326	(1.479–3.650)	0.000
N-stage	No vs. Yes	2.254	(1.533–3.313)	0.000
Histological grade	G1 vs. G2–G3	0.863	(0.652–1.142)	0.520
Clinical stage	I–II vs III–IV	2.741	(1.841–4.081)	0.000
14-3-3σ	Low vs. High	0.508	(0.316–0.816)	0.005
Multivariate analysis				
Age	<60 vs. ≥60	1.199	(0.750–1.917)	0.449
Gender	Male vs. Female	0.658	(0.348–1.241)	0.196
T-stage	T1+T2 vs. T3+T4	2.577	(1.605–4.150)	0.000
N-stage	No vs. Yes	0.755	(0.167–3.418)	0.715
Histological grade	G1 vs. G2–G3	0.621	(0.323–1.195)	0.154
Clinical stage	I–II vs. III–IV	3.042	(0.679–13.635)	0.146
14-3-3σ	Low vs. High	0.466	(0.251–0.866)	0.016

### SVM Classifier and ESCC Survival

**Figure 4:**
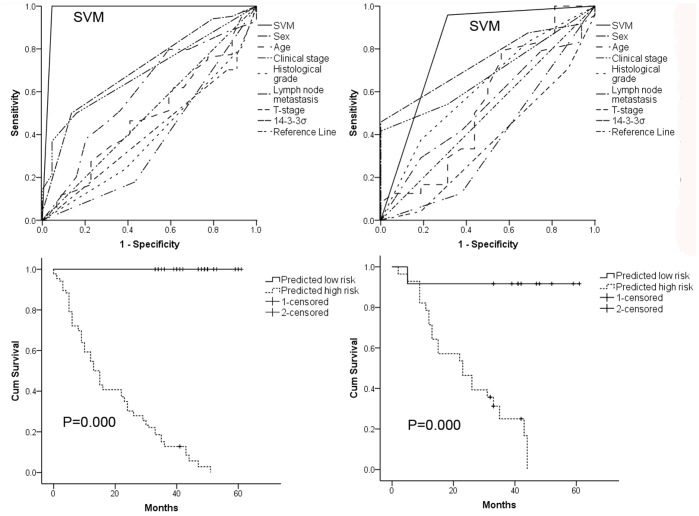
Receiver operating characteristic (ROC) curves and Kaplan-Meier survival estimates of evaluated ESCC patients from both (A, C) training and (B, D) validation cohorts. ROC curves for sex, age, T stage, histological grade, lymph node metastasis, clinical stage, 14-3-3σ and support vector machine (SVM) classifier as predictors for 5-year survival in training (A) and validation (B) cohorts. Kaplan-Meier survival estimates for low- and high-risk ESCC patients as defined by SVM classifier. Overall survival curves of evaluated patients in training (C) and validation (D) cohorts. Log-rank test used to calculate *P* values.

There were no statistically significant differences between the training cohort and the validation cohort in terms of 14-3-3σ immunostaining score or clinicopathological features including age, sex, clinical stage, histological grade, lymph node metastasis, T stage and survival (Table 4). The six clinicopathological features and 14-3-3σ expression were used for SVM analysis of the training data set to develop a SVM classifier. This comprised sex, age, T stage, histological grade, lymph node metastasis, clinical stage and 14-3-3σ expression as the optimal factors. The ROC curves of the SVM classifier, 14-3-3σ and other clinicopathological parameters are shown in figure 4A & 4B and the ROC curves of the SVM classifier produced the largest AUC both in the training and validation cohorts (0.98, 0.82, respectively), which was significantly greater than those of any of the prognostic factors used individually. The exclusion of 14-3-3σ from the SVM classifier with six signatures, however, led to a significant decrease of the AUCs from 0.82 to 0.63 (data not shown). The SVM classifier was significantly correlated with sex, clinical stage, lymph node metastasis and T stage. Based on the SVM classifier, 12 ESCC patients were defined as low risk and 28 patients as high risk in the validation cohort. The 5-year overall survival rates differed significantly between low- and high-risk ESCC patients (91.67% vs. 3.62%, log-rank test, χ^2^ = 16.175, *P* = 0.000, Figure 4D).

**Table 4:** Clinicopathological features of ESCC patients in the training and validation cohorts.

Features		Training cohort (128)		Validation cohort (40)		*P*
		No.	%	No.	%	
Sex	Male	94	73.4	31	77.5	0.607
	Female	34	26.6	9	22.5	
Age	Median	65		63		0.744
	Range	51–80		42–81		
Clinical stage	I–II	80	62.5	29	72.5	0.247
	III–IV	48	37.5	11	27.5	
Histological grade	G1	19	14.8	9	22.5	0.469
	G2	72	56.3	19	47.5	
	G3	37	28.9	12	30.0	
Lymph node metastasis	Median	0		0		0.329
	Range	0–14		0–14		
14-3-3σ	Median	4		4		0.953
	Range	0–12		0–12		
T-stage	T1+T2	23	18.0	9	22.5	0.527
	T3+T4	105	82.0	31	77.5	
Survival	Median	29		32		0.798
	Range	0–61		2–61		

## Discussion

Although the 5-year survival rate is only 3.1% for ESCC with metastasis, 18.5% for cancers with regional spread and 37.1% for localized primary cancers [46], it can be as high as 90–100% for CIS and intramucosal ESCC [4], [5]. This clearly demonstrates that early diagnosis of ESCC is one of the key determinants for long-term survival of ESCC. However, the lack of symptoms generated by early stage ESCC precludes its early detection. Biomarkers that permit identification of ESCC precursors may offer the best chance for ESCC control.

First and foremost, the present study demonstrated that dysregulation of 14-3-3σ expression occurred at pre-malignant stages during the multi-step development and progression of esophageal carcinogenesis. In terms of morphology, transformation of normal esophageal epithelium to malignant cells involves a series of pathological changes, i.e. basal cell hyper-proliferation, dysplasia, carcinoma in situ, invasive ESCC and distant metastasis [47]. The more severely diseased epithelium correlates with a higher risk of malignant transformation. A trend of decreasing 14-3-3σ protein expression was observed during precancerous lesions progression to ESCC. It is noteworthy that a significant difference was found between NEE and HGIN, NEE and ESCC, LGIN and ESCC. Previous studies indicated that at least 75% of severe dysplasia and carcinoma *in situ* progress to invasive ESCC [48]. In human epidermal keratinocytes, inactivation of 14-3-3σ caused immortalization, a fundamental feature of cancer cells [49]. Suppression of 14-3-3σ arises as malignant disease progresses from late atypical hyperplastic lesions, to ductal carcinoma in situ, and subsequently to invasive ductal carcinoma in breast cancer [50]. Consistently, one immortalized esophageal epithelial cell line NEC showed downregulation of 14-3-3σ. Together with the association of 14-3-3σ with ESCC precursor progression, our investigation indicates that 14-3-3σ has the potential to be a biomarker defining a subset of high-risk subjects predisposed to developing ESCC.

There have been conflicting reports concerning the role of 14-3-3σ in tumor formation and development although it has generally been regarded as a tumor suppressor. By sequestering cdc2-cyclin B1 complex in the cytoplasm, 14-3-3σ causes G_2_-M phase arrest which allows DNA damage repair and thus prevent genomic instability [51], [52]. Therefore, downregulation of 14-3-3σ may play a key role in carcinogenesis in several human malignancies [53]. On the other hand, overexpression of 14-3-3σ has also been documented in some cancers and both over- and under-expression of 14-3-3σ have been reported in the same type of cancer, such as ovarian cancer [29], [30], [54], ESCC [33], [42]. In prostate cancer, a significant downregulation of 14-3-3σ was found during the progression of normal prostatic epithelium to prostatic intraepithelial neoplasia and invasive cancer. However, islands of tumor cells with or without 14-3-3σ expression coexisted sometimes in the same specimen and a paradoxical higher level of 14-3-3σ expression was observed in adenocarcinomas with high Gleason scores compared with those with low Gleason scores, indicating that cells retaining 14-3-3σ expression may be selected during disease progression and treatment [26]. Indeed, an increased level of 14-3-3σ expression was found in drug (adriamycin)-selected breast cancer cell lines [55] and androgen-independent prostate cancer cell lines more resistant to mitoxantrone and adriamycin compared to androgen-dependent cell lines [56]. In accordance with these findings, one paclitaxel-resistant sub-line EC9706/PTX and one cisplatin-resistant sub-line EC9706/CDDP derived from the same parental cell line EC9706 showed higher levels of 14-3-3σ protein expression compared with immortalized NEC and ESCC cell lines. Furthermore, a high level of 14-3-3σ in patients at the advanced clinical stage and with lymph node metastasis did not predict good clinical outcome contrasting sharply with its role in ESCC patients at early clinical stage and negative lymph node metastasis (Figure 3). Taken together, we propose that recovery from 14-3-3σ suppression could enhance progression of later stage ESCC and contribute to paclitaxel/cisplatin-resistance during therapeutic intervention. In cancers with lymph node metastases, elevated expression of 14-3-3σ was frequently observed in ovarian cancer [54], gastric cancer [57], endometrial cancer [58], pancreatic cancer [59] and nasopharyngeal carcinoma [31]. A study from Japan reported that elevated nuclear expression of 14-3-3σ in 248 ESCC patients was significantly correlated with depth of invasion, clinical stage and lymphatic invasion whereas there was no association between cytoplasmic expression of 14-3-3σ and clinical factors [42]. In sharp contrast, predominant cytoplasmic staining of 14-3-3σ was observed, and notably, decreased or complete loss of 14-3-3σ expression was significantly correlated with lymph node metastasis in another study (148 samples) using ESCC samples from China [33]. In our study, 14-3-3σ protein was mainly located in the cytoplasmic and plasma membrane and less frequently in the nuclei, in particular in late stage ESCC. In addition, the decreased expression of 14-3-3σ that correlated with histological grade by IHC analysis was inconsistent with Western blot results of a correlation with clinical stage (Figure 1B & C). The precise reason for these discrepancies is unknown but possible explanations include geographical location, hereditary factors, environmental factors, technical issues in sample processing, disease stage, etc. In the current study, the samples used for Western blot were fresh frozen from Linzhou Cancer Hospital, Henan (a well-known high-incidence area for ESCC) whereas the samples for IHC analysis were formalin-fixed tissue from Huaihe Hospital, Henan and TMA from Shanghai (low-incidence areas for ESCC) and this may affect the 14-3-3σ expression pattern. Clearly more studies are needed to elucidate the functions of 14-3-3σ in the progression of specific cancers.

Current clinical staging systems for ESCC are of limited value in prognosis and novel molecular biomarkers with prognostic value are urgently required. Since chemotherapy or chemoradiation is effective only in around 50% of patients [3], it is important for patients with good prognosis to avoid potential overtreatment so as to escape treatment toxicities. Assessment of prognosis on the basis of molecular characteristics would help inform decisions and tailor therapy to ESCC individuals so as to achieve the best possible outcome. Our survival analysis revealed that downregulation of 14-3-3σ was significantly correlated with poor prognosis of ESCC. Thus, patients with reduced expression of 14-3-3σ had a significantly lower 5-year survival rate relative to ESCC patients with a high level of 14-3-3σ expression. Multivariate analysis found that 14-3-3σ was an independent prognostic factor for ESCC. Other studies form China support the current findings [33], but a study form Japan reported that overexpression of 14-3-3σ in the nucleus was a poor prognosis factor [42]. Esophageal carcinogenesis is a complex dynamic biological process involving a myriad of molecular alternations in a multi-stage evolution. It seems unlikely therefore that a single gene expression could suffice to predict the prognosis of ESCC. As such, SVM was used to build a reliable ESCC classifier on the basis of clinicopathological features and 14-3-3σ expression to improve the accuracy of prognostication. The predictive accuracy of our ESCC classifier incorporating sex, age, T stage, histological grade, lymph node metastasis, clinical stage and 14-3-3σ expression was better than any individual component. The exclusion of 14-3-3σ from SVM classifier greatly decreased the predictive accuracy, indicating that 14-3-3σ made a substantial contribution to our current SVM classifier for ESCC prognosis prediction.

In summary, our study reveals that downregulation of 14-3-3σ arises early in the onset of ESCC and is a potential biomarker for early detection of high-risk subjects and for early diagnosis of ESCC and an independent prognosis factor of ESCC. A seven-feature SVM classifier showed powerful predictive utility for ESCC overall survival. In view of the conflicting reports on the clinical relevance and prognostic value of 14-3-3σ in ESCC, more research is warranted to define its role in the multistage carcinogenesis of ESCC.

## Supporting Information

Table S1 Summary of clinicopathological features of patients.
(DOC)
