# Supplementary material for: Downregulation of 14-3-3σ Correlates with Multistage Carcinogenesis and Poor Prognosis of Esophageal Squamous Cell Carcinoma
Source: PLoS One. 2014 Apr 17;9(4):e95386. doi: 10.1371/journal.pone.0095386 (PMC3990633; doi:10.1371/journal.pone.0095386)
Supplement: Table S1 — Summary of clinicopathological features of patients. (DOC) [file pone.0095386.s001.doc]

Table S1 Summary of clinicopathological features of patients.

| Variables | Biopsy | Fresh ESCC | Archival ESCC |
| --- | --- | --- | --- |
| Gender |  |  |  |
| Male | 72 | 52 | 125 |
| Female | 38 | 28 | 43 |
| Age (years) | | | |
| ＜60 | 44 | 31 | 55 |
| ≥60 | 66 | 49 | 113 |
| Tissue type | | | |
| Normal esophageal epithelium | 41 | - | - |
| Low grade intraepithelial neoplasia | 37 | - | - |
| High grade intraepithelial neoplasia | 32 | - | - |
| Histological grade | | | |
| Well-differentiated | - | 27 | 28 |
| Moderately differentiated | - | 41 | 91 |
| Poorly differentiated | - | 12 | 49 |
| T stage | | | |
| T1-T2 | - | 23 | 32 |
| T3-T4 | - | 35 | 118 |
| Lymph node metastasis |  |  |  |
| Yes | - | 37 | 67 |
| No | - | 29 | 97 |
| Clinical stage | | | |
| Ⅰ | - | 20 | 10 |
| Ⅱ | - | 20 | 87 |
| Ⅲ | - | 20 | 55 |
| Ⅳ | - | 20 | 4 |

Note: Low grade intraepithelial neoplasia comprises mild and/or moderate dysplasia; High grade intraepithelial neoplasia comprises severe dysplasia and/or carcinoma in situ.
